# Nonlinear dose–response evaluation of RenalMax supplementation in broiler chickens

**DOI:** 10.1016/j.vas.2026.100822

**Published:** 2026-08-15

**Authors:** Morteza Asghari-Moghadam, Hamid-Reza Behboodi, Mehran Mehri

**Affiliations:** a Department of Animal Sciences, Faculty of Agriculture, University of Zabol, Sistan, 98613-35856, Iran; b Department of Animal and Poultry Physiology, Faculty of Animal Science, Gorgan University of Agricultural Science and Natural Resources, Gorgan, 49138-15739, Iran; c Research and Development Department, Makian Rooyesh Darou Co. Gorgan, Iran

**Keywords:** Antioxidant potency, Creatinine, Uric acid, Modeling, Phytogenic

## Abstract

This study evaluated the effects of RenalMax, a phytogenic blend containing nettle extract, sorbitol, ammonium chloride, and vitamins (B1, B2, B6, and C), on growth performance, serum biochemical variables, antioxidant status, and mineral metabolism in broiler chickens. A total of 400 one-day-old male Ross 308 broilers were randomly assigned to four treatments receiving 0, 0.25, 0.50, or 1.00 mL RenalMax/L drinking water, with five replicate pens of 20 birds per treatment, and were reared for 42 d RenalMax supplementation increased body weight gain and feed intake (P < 0.05), with the greatest response observed at 0.50 mL/L. Serum uric acid, creatinine, and blood urea nitrogen concentrations decreased (P < 0.001), whereas albumin and total protein concentrations increased. Supplementation was also associated with lower serum triglyceride, total cholesterol, low-density lipoprotein, alanine aminotransferase, aspartate aminotransferase, alkaline phosphatase, and malondialdehyde concentrations, together with higher high-density lipoprotein, total antioxidant capacity, glutathione peroxidase activity, and serum calcium and phosphorus concentrations (P < 0.05). Principal component analysis distinguished treatment groups based on the combined variation of growth performance, serum biochemical, antioxidant, and mineral variables. Dose-response analyses using polynomial and segmented regression models identified nonlinear responses for several variables, with model-estimated optima generally occurring between 0.5 and 0.6 mL/L within the evaluated supplementation range. Overall, RenalMax supplementation was associated with favorable changes in growth performance and multiple serum biochemical, antioxidant, and mineral-related variables in broiler chickens, although the estimated optimal supplementation level should be confirmed under commercial production conditions.

## Highlights

RenalMax improved growth performance and feed intake in broiler chickens.
The optimal dose (0.50 mL/L) enhanced metabolic efficiency and reduced fat deposition.
RenalMax improved lipid profile by lowering TG, LDL, and cholesterol while increasing HDL.
Renal and hepatic function markers improved, indicating enhanced organ health.
Antioxidant capacity increased, while lipid peroxidation (MDA) was significantly reduced.

## 1 Introduction

The increasing restriction on the use of antibiotic growth promoters has stimulated interest in phytogenic additives and functional nutrients as sustainable strategies to improve poultry health and productivity. Phytogenic compounds contain numerous bioactive constituents with antioxidant, anti-inflammatory, and antimicrobial properties that may enhance growth performance, nutrient utilization, and physiological homeostasis in poultry (Flees et al., 2021b; Wang et al., 2021; Biswas & Kim, 2025). Their efficacy may be further enhanced when combined with complementary nutrients that target multiple physiological pathways involved in metabolism and oxidative balance.

Among phytogenic ingredients, *Urtica dioica* (stinging nettle) has received considerable attention because of its high concentration of polyphenols and flavonoids with antioxidant activity. Experimental studies have shown that nettle extract can reduce oxidative stress and modify serum biochemical variables associated with liver and kidney function under different physiological challenges (Adejuwon et al., 2014; Buchko et al., 2019, 2023; Muceniece et al., 2025). Likewise, vitamins C and B-complex participate in antioxidant defense, energy metabolism, and cellular biochemical reactions, while sorbitol and ammonium chloride have traditionally been incorporated into poultry formulations to support metabolic and urinary functions. Collectively, these components may exert complementary physiological effects, although their combined responses in broiler chickens have not been adequately investigated.

Most previous studies have evaluated individual phytogenic extracts or single nutrients, making it difficult to determine whether combining phytogenic and nutritional components provides additional biological benefits. Consequently, information remains limited regarding the integrated effects of multi-component formulations on growth performance, serum biochemical variables, antioxidant status, lipid metabolism, and mineral metabolism in broiler chickens. Evaluating the complete formulation rather than its individual ingredients is particularly relevant because commercial products are administered as mixtures in which interactions among components may influence the overall biological response.

Therefore, the objective of the present study was to evaluate the effects of RenalMax, a phytogenic formulation containing nettle extract, sorbitol, ammonium chloride, and vitamins B_1_, B_2_, B_6_, and C, on growth performance, serum biochemical variables, antioxidant status, lipid metabolism, and mineral metabolism in broiler chickens. In addition, nonlinear dose-response analyses were performed to characterize the response patterns across supplementation levels and to estimate the supplementation range associated with the most favorable biological responses under the experimental conditions.

## 2 Materials and methods

### 2.1 Ethics statement

This study protocol adheres to the guidelines established by the Iranian Council of Animal Care and it has been approved by the Research Animal Ethics Committee (AEUOZ-2012|UP-2020-BR-21) at the University of Zabol.

### 2.2 Preparation of the Renalmax

RenalMax is a proprietary commercial formulation. While the exact proportions of individual ingredients cannot be disclosed because of commercial confidentiality, the active ingredients and phytochemical characterization of the standardized nettle extract are provided to facilitate scientific interpretation. The RenalMax mixture, a blend of (nettle extract, sorbitol, ammonium chloride, vitamin B_1_, vitamin B_2_, vitamin B_6_ and vitamin C), was carefully prepared to ensure a consistent final concentration of its components, and its physical and chemical properties and composition of *Urtica dioica L*. solution extract were shown in Table 1.

**Table 1:** Composition of *Urtica dioica L*. solution extract and physico-chemical properties of RenalMax solution.

*Urtica dioica L*. solution extract	Amount
Total phenolics content (mg/g)	3.45
Chlorogenic acid (μg/g)	301
Cafeic acid (μg/g)	171
Kaempferl (μg/g)	301
Quercetin (μg/g)	303
RenalMax solution	Amount
Specific Gravity (g/ml)	0.9912
Refractive Index	1.4393
pH	5.60
Density	1.004
Assay (Coffeoylmalic acid and chlorogenic acid)	0.0935%
Average Volume (ml)	1000 ml

### 2.3 Birds, housing, and experimental design

A total of 400 one-day-old male broiler chicks (Ross 308) were obtained from a local hatchery (initial body weight: 39.4 ± 2.15 g). Birds were randomly assigned to four dietary treatments with five replicate pens per treatment and 20 birds per pen. Each pen measured 2.0 × 1.5 m (3.0 m²), resulting in an initial stocking density of approximately 6.7 birds/m². Birds were housed on a concrete floor covered with fresh wood shavings, and each pen was equipped with one bell-shaped drinker and one feeder. Environmental conditions, including temperature and lighting programs, were managed according to commercial Ross 308 recommendations. Drinking water was the method of treatment administration; control (no RenalMax), and RenalMax concentrations of 0.25, 0.50, and 1.00 mL/L were added on alternating days, beginning on day 10, as per the manufacturer's guidelines. The initiation of supplementation on day 10 was selected because birds enter a period of accelerated growth and increasing metabolic demand after the early brooding phase. On each supplementation day, RenalMax was freshly diluted in drinking water and provided continuously throughout the day. Fresh treated water was prepared and supplied according to bird age and water consumption to maintain uniform exposure. The birds had continuous access to food and water, receiving a commercial diet (Table 2) for the duration of the experiment, which was formulated accoding to Ross 308 breeder guidelines (Aviagen, 2022). A 33 °C temperature was used for a week, after which it was lowered by 2 °C per week to 22 °C. A continuous lighting protocol was used for 3 days, then changed to a 16L:8D cycle.

**Table 2:** Composition of basal diet.

Ingredient	Amount (%)		
	Starter	Grower	Finisher
Corn, Grain	52.50	49.06	52.50
Wheat, Red W.	20.61	15.00	20.61
Soybean Meal	18.98	26.75	18.98
Corn Gluten Meal	2.48	3.73	2.48
Limestone	1.44	1.42	1.44
DCP	1.31	1.47	1.31
Soybean Oil	1.00	1.09	1.00
NaHCO_3_	0.60	0.34	0.60
L-Lysine HCl	0.30	0.29	0.30
Vitamin Premix^1^	0.25	0.25	0.25
Mineral Premix^2^	0.25	0.25	0.25
DL-Methionine	0.15	0.20	0.15
NaCl	0.10	0.10	0.10
L-Thr	0.04	0.04	0.04
Nutrient specifications (analyzed)			
AME (kcal/kg)	2950	2950	2983
CP (%)	23.7	21.8	18.2
SID Lys (%)	1.20	1.10	0.91
SID M+C (%)	0.86	0.79	0.66
SID Thr (%)	0.76	0.69	0.57
SID Met (%)	0.56	0.51	0.41
*Ca* (%)	1.05	0.95	0.91
Available P (%)	0.50	0.45	0.40
DEB (mEq/kg)^3^	250	250	250

¹Vitamin premix supplied the following per kilogram of diet: vitamin A (trans-retinyl acetate), 12,000 IU; vitamin D₃ (cholecalciferol), 5000 IU; vitamin E (DL-α-tocopheryl acetate), 50 mg; vitamin K₃, 3 mg; thiamine (vitamin B₁), 3 mg; riboflavin (vitamin B₂), 8 mg; niacin, 50 mg; pantothenic acid, 15 mg; pyridoxine (vitamin B₆), 4 mg; biotin, 0.15 mg; folic acid, 2 mg; vitamin B₁₂ (cyanocobalamin), 0.02 mg; choline chloride, 500 mg.

2 Provided the following per kilogram of diets: Mn (from MnSO4, H 2O), 40 mg; Zn (from ZnO), 40 mg; Fe (from FeSO4·7H2O), 20 mg; Cu (from CuSO4, 5H2O), 4 mg; I [from *Ca* (IO3)2H2O], 0.64 mg; Se (from sodium selenite), 0.08 mg.

3 Dieary electrolyte balance (Na^+^+ K^+^- Cl^-^).

### 2.4 Performance parameters

To calculate body weight gain, feed intake, and feed conversion ratio, birds were weighed on a pen basis at hatch and 7, 14, 21, 35, and 42 days old. Mortality was recorded daily throughout the experimental period, including the date of occurrence and suspected cause of death. The corrected feed conversion ratio was calculated considering mortality according to standard broiler performance evaluation procedures. On day 42, two birds per pen (*n* = 10 birds per treatment) were selected, euthanized, and dissected to assess the relative weights of their internal organs. The breast, thigh, abdominal fat, liver, and heart were excised and weighed. The relative weights of tissues (breast meat yield: BMY and thigh meat yield: TMY) and organs were expressed as a percentage of live body weight.

### 2.5 Blood collection

At 21 and 42 days of age, two birds from each pen were randomly selected for blood collection. To minimize the influence of circadian variation on blood biochemical parameters, all blood samples were collected at the same time of day (08:00–10:00 h) before routine feeding and management activities. Blood samples were collected from the brachial vein into sterile tubes and allowed to clot at room temperature before serum separation by centrifugation at 3000 × g for 10 min. The obtained serum samples were stored at −20 °C until biochemical analyses.

### 2.6 Biochemical analyses

Total protein (TP) concentration was quantified by the biuret method, whereas serum albumin was measured using the bromocresol green (BCG) method with commercial kits (Pars Azmun, Tehran, Iran). Absorbance was recorded at 630 nm with a spectrophotometer, and serum globulin was determined as the difference between total protein and albumin concentrations (Kumar et al., 2005). Serum concentrations of cholesterol, high-density lipoprotein (HDL), triglycerides (TG), and blood urea nitrogen (BUN) were determined spectrophotometrically using a microplate reader (Stat Fax 3200; Awareness Technology Inc., Palm City, FL, USA) with Pars Azmun diagnostic kits. Low-density lipoprotein (LDL) was calculated according to the equation described by Taherpour et al. (2009):

LDL=Totalcholesterol−HDL−TG/5

### 2.7 Hepatic enzyme activity

Alanine aminotransferase (ALT) and aspartate aminotransferase (AST) activities were assessed according to the procedure of Reitman and Frankel (1957), in which 0.3 ml of serum was combined with 300 ml of reagent from Pars Azmun. Alkaline phosphatase (ALP) was analyzed by the DEA method, using 20 µl of serum mixed with 1 ml of reagent and incubated at 37 °C (Sun et al., 2018). The absorbance changes for ALT, AST, and ALP were monitored at 340 nm.

### 2.8 Antioxidant assay

Plasma antioxidant indices, including total antioxidant capacity (TAC), total superoxide dismutase (T-SOD), and glutathione peroxidase (GPX), were determined colorimetrically with a Biomate 5 spectrophotometer (Thermo Electron Corporation, Rochester, NY, USA) using commercial assay kits (Randox Laboratories Ltd., Crumlin, UK) (Baser et al., 2015; Liu et al., 2017). Lipid peroxidation was evaluated as malondialdehyde (MDA) concentration via the thiobarbituric acid reactive substances (TBARS) method. For this assay, 0.5 ml of serum was mixed with 1 ml of trichloroacetic acid, centrifuged at 5000 rpm for 10 min, and 0.5 ml of the supernatant was subsequently reacted with 0.5 ml of 2-thiobarbituric acid solution. The mixture was incubated in a boiling water bath at 100 °C for 10 min, cooled, and the absorbance was read at 532 nm (Wang et al., 2008).

### 2.9 Statistical analysis

All data were analyzed using SAS software (version 9.4; SAS Institute Inc., Cary, NC, USA). The pen was considered the experimental unit for all statistical analyses (n = 5 pens per treatment). For carcass traits and all serum biochemical, hepatic enzyme, antioxidant, and mineral variables, two birds were sampled from each pen, and the mean of the two birds was used as a single pen observation for statistical analysis. A one-way analysis of variance (ANOVA) was performed with RenalMax inclusion level as the main fixed effect. Polynomial contrasts were applied to assess linear and quadratic dose–response trends. Model assumptions (normality and homogeneity of variance) were verified using residual diagnostics. To characterize the dose–response relationships and estimate the supplementation level associated with the most favorable responses, parabolic polynomial (PP), two-slope linear (LL), and two-slope quadratic–linear (QL) regression models were fitted following Khosravi et al. (2016). Model performance was compared using the coefficient of determination (R²), residual standard deviation (Sy.x), root mean square error (RMSE), and corrected Akaike Information Criterion (AICc), and the model with the best overall fit was selected for each response variable.

$$S_{y.x}=\sqrt{\frac{SS}{df}}$$

$$AIC=n.\ln\left(\frac{RSS}{n}\right)+2k$$

$$RSS=\;\sum_{i=1}^{n}{\left(y_{i}-\;\hat{y_{i}}\right)}^{2}=residual\;sum\;of\;squares$$

n=numberofobservations

k=numberofestimatedparametersinthemodel(includingtheintercept)

Principal component analysis (PCA) was used to explore multivariate patterns among serum biochemical, hepatic enzyme, antioxidant, and performance variables. The first two principal components were retained to visualize clustering and interpret variable contributions to the observed variance. All results are presented as means ± standard error of the mean (SEM), where SEM reflects variation among pen means. Data visualization was performed using GraphPad Prism (version 9.0) and SAS (2002).

## 3 Results

### 3.1 Growth performance and carcass characteristics

Average daily water consumption was recorded at the pen level throughout the experimental period. Mean water consumption was approximately 7, 14, 16–18, 20–22, 25–28, and 32–35 L/pen/day during weeks 1 to 6, respectively. Based on these values, estimated RenalMax intake increased proportionally with age and supplementation level. For example, at 0.50 mL/L, estimated daily intake increased from approximately 3.5 mL/pen/day during week 1 to approximately 16.8 mL/pen/day during week 6.

RenalMax supplementation affected growth performance (Table 3). Feed intake increased significantly (ANOVA P < 0.0001) and exhibited both significant linear (P-linear < 0.0001) and quadratic (P-quadratic < 0.0001) responses, with the highest mean value observed at 0.50 mL/L. Body weight gain also differed among treatments (ANOVA P < 0.0001) and showed significant linear (P-linear = 0.019) and quadratic (P-quadratic < 0.0001) responses. Feed conversion ratio differed among treatments (ANOVA P = 0.018), although neither the linear (P = 0.127) nor quadratic (P = 0.206) contrast was significant. Dressing percentage differed among treatments (ANOVA P < 0.0001) with significant linear and quadratic responses (both P < 0.0001), although the numerical differences among treatments were small. Thigh meat yield (P = 0.206) and breast meat yield (P = 0.543) were unaffected by supplementation.

**Table 3:** Effects of RenalMax on performance and dressing of broiler chickens.

Response	RenalMax (ml.L^–1^)			SEM			Probability		
	0.0	0.25	0.50	1.00		ANOVA	Control *vs.* RenalMax	Linear	Quadratic
Feed intake (g/b/d)	118	121	125	121	0.40	<.0001	<.0001	<.0001	<.0001
Gain (g/b/d)	66.1	67.1	70.4	66.7	0.42	<.0001	0.001	0.019	<.0001
Feed conversion ratio	1.78	1.81	1.77	1.82	0.01	0.018	0.223	0.127	0.206
Dressing (%)	71.5	71.6	70.9	71.5	0.01	<.0001	<.0001	<.0001	<.0001
Thigh meat yield (%)	21.9	22.0	21.9	21.9	0.01	0.206	0.133	0.745	0.129
Breast meat yield (%)	23.9	24.0	23.9	23.9	0.01	0.543	0.195	0.392	0.470
Fat pad (%)	2.03	2.06	1.41	2.04	0.01	<.0001	<.0001	<.0001	<.0001
Heart (%)	0.65	0.68	0.58	0.66	0.03	0.078	0.769	0.574	0.364
Liver (%)	2.81	2.35	2.52	2.69	0.05	0.001	0.001	0.513	<.0001

Abdominal fat percentage differed markedly among treatments (ANOVA P < 0.0001) and exhibited significant linear (P-linear < 0.0001) and quadratic (P-quadratic < 0.0001) responses, with the lowest value observed at 0.50 mL/L. Relative liver weight was also affected (ANOVA P = 0.001) and showed a significant quadratic response (P-quadratic < 0.0001) but no significant linear trend (P-linear = 0.513). Heart weight was not affected (P = 0.078).

Mortality remained low throughout the study and did not differ significantly among treatments (P > 0.05). Mortality rates were 9.0, 8.0, 5.0, and 6.0% for the control, 0.25, 0.50, and 1.00 mL/L groups, respectively.

### 3.2 Renal function and nitrogen metabolism

RenalMax supplementation affected several serum nitrogen metabolites (Table 4). Uric acid differed among treatments on day 21 (ANOVA P = 0.001) and day 42 (P = 0.010). A significant quadratic response was detected on both sampling days (P = 0.001 and P = 0.003, respectively), whereas the linear response was significant only on day 21 (P = 0.066 on day 21; P = 0.836 on day 42).

**Table 4:** Effects of RenalMax on blood nitrogen constitutes of broiler chickens at 21 and 42 d of age.

Response	RenalMax (ml.L^–1^)				SEM	Probability			
	0.0	0.25	0.50	1.00		ANOVA	Control *vs.* RenalMax	Linear	Quadratic
UA_21_ (mg/dL)	3.15	2.49	2.64	2.83	0.09	0.001	0.001	0.066	0.001
UA_42_ (mg/dL)	3.33	3.07	3.18	3.27	0.05	0.010	0.017	0.836	0.003
CRT_21_ (mg/dL)	1.88	1.26	1.53	1.38	0.09	0.002	0.001	0.011	0.029
CRT_42_ (mg/dL)	1.87	1.48	1.59	1.65	0.04	<0.001	<.0001	0.007	<.0001
BUN_21_ (mg/dL)	0.65	0.51	0.54	0.62	0.03	0.008	0.011	0.651	0.001
BUN_42_ (mg/dL)	0.75	0.61	0.63	0.67	0.01	<0.001	<.0001	0.001	<.0001
ALB (g/dL)	1.67	2.05	1.77	1.43	0.15	0.059	0.657	0.143	0.027
TP (g/dL)	4.46	5.70	4.79	5.07	0.34	0.104	0.081	0.547	0.177

UA: uric acid, CRT: creatinine, BUN: blood urea nitrogen, ALB: albumin, TP: total protein.

Serum creatinine differed among treatments on day 21 (P = 0.002) and day 42 (P < 0.001). Significant linear (P = 0.011 and 0.007) and quadratic (P = 0.029 and P < 0.0001) responses were observed.

Blood urea nitrogen differed among treatments on day 21 (P = 0.008) and day 42 (P < 0.001). Day-21 values exhibited only a quadratic response (P = 0.001), whereas both linear (P = 0.001) and quadratic (P < 0.0001) responses were significant on day 42.

Albumin exhibited a significant quadratic response (P = 0.027) despite the overall ANOVA not reaching significance (P = 0.059). Total protein was unaffected by treatment (ANOVA P = 0.104).

### 3.3 Serum lipid profile and glucose

RenalMax supplementation affected all lipid variables and serum glucose (Table 5). Triglycerides, VLDL, total cholesterol, LDL, HDL, and glucose all differed among treatments (all ANOVA P < 0.001). Significant linear and quadratic dose-response effects were detected for all variables (all P ≤ 0.001), with HDL increasing while triglycerides, VLDL, LDL, cholesterol, and glucose generally decreased as supplementation increased to intermediate concentrations.

**Table 5:** Effects of RenalMax on glucose and lipid constitutes in serum of broiler chickens.

Response	RenalMax (ml.L^–1^)				SEM	Probability			
	0.0	0.25	0.50	1.00		Model	Control *vs.* RenalMax	Linear	Quadratic
TG (mg/dL)	92.8	81.2	70.1	81.1	2.19	<0.001	<.0001	0.001	<.0001
VLDL (mg/dL)	18.6	16.2	14.0	16.2	0.44	<0.001	<.0001	0.001	<.0001
HDL (mg/dL)	47.9	70.9	83.8	65.9	2.05	<0.001	<.0001	<.0001	<.0001
LDL (mg/dL)	131	88.4	48.5	84.9	3.37	<0.001	<.0001	<.0001	<.0001
COL (mg/dL)	198	176	146	167	2.54	<0.001	<.0001	<.0001	<.0001
GLU (mg/dL)	289	279	242	282	3.31	<0.001	<.0001	0.001	<.0001

TG: triglyceride, VLDL: very low-density lipoprotein, HDL: high-density lipoprotein, LDL: low-density lipoprotein, COL: cholesterol, GLU: glucose.

### 3.4 Hepatic enzyme activities

Activities of AST, ALT, and ALP were affected by RenalMax supplementation (all ANOVA P < 0.001). Significant linear (all P < 0.0001) and quadratic (all P < 0.0001) responses were observed for each enzyme activity (Table 6), with the lowest mean values generally occurring at the intermediate supplementation level.

**Table 6:** Effects of RenalMax on hepatic enzyme activities in serum of broiler chickens.

Response	RenalMax (ml.L^–1^)				SEM	Probability			
	0.0	0.25	0.50	1.00		Model	Control *vs.* RenalMax	Linear	Quadratic
AST (U/L)	296	279	245	278	3.20	<0.001	<.0001	<.0001	<.0001
ALT (U/L)	25.1	21.3	11.7	17.0	0.82	<0.001	<.0001	<.0001	<.0001
ALP (U/L)	1958	1566	818	1352	46.9	<0.001	<.0001	<.0001	<.0001

AST: aspartate aminotransferase, ALT: alanine aminotransferase, ALP: alkaline phosphatase.

### 3.5 Antioxidant variables

RenalMax supplementation affected all antioxidant variables (Table 7). Total antioxidant capacity, glutathione peroxidase, superoxide dismutase, and malondialdehyde differed among treatments (all ANOVA P < 0.001). Significant linear and quadratic responses were detected for all antioxidant indices (all P < 0.0001). TAC, GPX, and SOD reached their greatest values at intermediate supplementation levels, whereas MDA exhibited the opposite pattern. Table 8.

**Table 7:** Effects of RenalMax on antioxidant system of broiler chickens.

Response	RenalMax (ml.L^–1^)				SEM	Probability			
	0.0	0.25	0.50	1.00		Model	Control *vs.* RenalMax	Linear	Quadratic
TOC (nmol/mL)	3.45	4.63	7.21	4.95	0.31	<0.001	<.0001	<.0001	<.0001
GPX (U/g Hg)	269	314	412	327	9.47	<0.001	<.0001	<.0001	<.0001
SOD (U/g Hb)	2790	3050	3925	3302	62.3	<0.001	<.0001	<.0001	<.0001
MDA (nmol/mL)	4.13	3.15	2.09	3.18	0.09	<0.001	<.0001	<.0001	<.0001

TOC: total antioxidant capacity, GPX: glutathione peroxidase, SOD: superoxide dismutase, MDA: malondialdehyde.

**Table 8:** Effects of RenalMax on calcium (*Ca*) and phosphorus (P) in serum of broiler chickens.

Response	RenalMax (ml.L^–1^)				SEM	Probability			
	0.0	0.25	0.50	1.00		Model	Control *vs.* RenalMax	Linear	Quadratic
*Ca* (mg/dL)	6.57	17.3	18.9	12.2	1.41	<0.001	<0.001	0.009	<0.001
P (mg/dL)	16.5	21.4	23.2	15.9	1.63	0.014	0.064	0.951	0.002

### 3.6 Serum mineral concentrations

Serum calcium differed among treatments (ANOVA P < 0.001) and exhibited both significant linear (P = 0.009) and quadratic (P < 0.001) responses. Serum phosphorus also differed among treatments (ANOVA P = 0.014) and exhibited a significant quadratic response (P = 0.002), whereas the linear trend was not significant (P = 0.951).

### 3.7 Principal component analysis

Principal component analysis summarized the multivariate response of broilers to RenalMax supplementation (Table 9). Variables associated with serum lipid profile (TG, VLDL, LDL, cholesterol), hepatic enzymes (AST, ALT, ALP), oxidative stress (MDA), antioxidant variables (TAC, GPX, SOD), and feed intake showed the greatest absolute loadings on the first principal component. The second principal component was primarily associated with serum uric acid, creatinine, blood urea nitrogen, albumin, and total protein, whereas the third principal component was mainly influenced by RenalMax supplementation level and feed conversion ratio.

**Table 9:** Component loadings and uniqueness of performance, biochemical and oxidative stress markers with varimax rotation.

Items	Principal Component (PC)			Uniqueness
	1	2	3	
RenalMax	−0.5224	0.0827	−0.78,573	0.1029
UA_21_	0.3121	0.8673	−0.01,764	0.1501
UA_42_	0.2458	0.8490	−0.15,897	0.1935
CRT_21_	0.1519	0.6435	0.51,312	0.2996
CRT_42_	0.3798	0.7863	0.33,286	0.1267
BUN_21_	0.0613	0.8457	0.03,421	0.2799
BUN_42_	0.4701	0.7110	0.10,212	0.2630
ALB	−0.0892	−0.5951	0.67,312	0.1848
TP	−0.2763	−0.7551	0.34,976	0.2311
TG	0.9084	0.2848	0.06,810	0.0891
VLDL	0.8680	0.2973	0.14,346	0.1377
HDL	−0.8214	−0.4517	−0.02,887	0.1204
LDL	0.9205	0.3165	0.10,865	0.0407
COL	0.9341	0.2497	0.13,712	0.0463
GLU	0.8735	0.1740	−0.32,398	0.1017
AST	0.9237	0.2396	−0.11,279	0.0767
ALT	0.9332	0.0746	0.16,314	0.0970
ALP	0.9619	0.1835	0.08,478	0.0340
TOC	−0.9274	−0.2026	0.17,029	0.0699
GPX	−0.9368	−0.2170	0.15,453	0.0513
SOD	−0.9575	−0.0940	−0.00,508	0.0743
MDA	0.9105	0.3497	−0.00,211	0.0487
FI	−0.8661	−0.4042	−0.10,251	0.0760
G	−0.8340	−0.1539	0.42,603	0.0993
FCR	0.2860	−0.1974	−0.81,026	0.2227

UA: uric acid (d 21 and 42), CRT: creatinine (d 21 and 42), BUN: blood urea nitrogen (d 21 and 42), ALB: albumin, TP: total protein, TG: triglyceride, VLDL: very low-density lipoprotein, HDL: high-density lipoprotein, LDL: low-density lipoprotein, COL: cholesterol, GLU: glucose, AST: aspartate transaminase, ALT: alanine transaminase, ALP: alkaline phosphatase, TOC: total antioxidant capacity, GPX: gluthatione peroxidase, SOD: superoxide dismutase, MDA: amalodialdehyde, FI: feed intake, G: body weight, FCR: feed conversion ratio.

### 3.8 Dose–response modeling

Dose–response analyses demonstrated nonlinear responses for most measured variables (Table 10). Model comparison using R², *S*_y.x_, and AIC showed that LL model provided the best overall fit for most serum biochemical, lipid, hepatic enzyme, antioxidant, and oxidative stress variables. The PP model provided the best fit for calcium, phosphorus, and HDL, whereas QL and LL performed similarly for uric acid, blood urea nitrogen, and creatinine. Across response variables, the estimated optima generally occurred within the range of approximately 0.4 to 0.7 mL/L RenalMax. Because only four supplementation levels were experimentally evaluated, these estimates should be interpreted as model-derived predictions within the tested range rather than definitive biological optima.

**Table 10:** Comparison of dose–response models fitted to RenalMax supplementation for each response variable.

Trait	Selected model	R²	*S*_y.x_	AIC
UA21	Two-slope QL / Two-slope LL	0.774	0.153	−65.38
UA42	Two-slope QL / Two-slope LL	0.646	0.086	−88.38
ALB	Two-slope LL	0.741	0.181	−58.62
ALP	Two-slope LL	0.934	125.6	203.10
ALT	Two-slope LL	0.851	2.263	42.50
AST	Two-slope LL	0.907	6.694	85.87
BUN21	Two-slope QL / Two-slope LL	0.571	0.0476	−112.0
BUN42	Two-slope QL / Two-slope LL	0.788	0.0282	−132.9
Ca	Parabolic Polynomial	0.848	2.097	37.04
CHOL	Two-slope LL	0.947	4.829	72.80
CRT21	Two-slope QL / Two-slope LL	0.514	0.235	−48.17
CRT42	Two-slope QL / Two-slope LL	0.869	0.0619	−101.5
GLU	Two-slope LL	0.807	9.660	100.5
GPX	Two-slope LL	0.903	18.76	127.1
HDL	Parabolic Polynomial	0.906	4.502	67.59
LDL	Two-slope LL	0.957	6.840	86.74
MDA	Two-slope LL	0.935	0.213	−51.99
P	Parabolic Polynomial	0.898	1.758	29.99
TP	Two-slope QL	0.690	0.325	−35.09
SOD	Two-slope LL	0.861	171.6	215.6
TAC	Two-slope LL	0.866	0.591	−11.20
TG	Two-slope LL	0.870	3.596	61.01
VLDL	Two-slope LL	0.814	0.816	1.68

R², coefficient of determination; *S*_y.x_, standard error of regression; AIC, Akaike information criterion. UA, uric acid; BUN, blood urea nitrogen; CRT, creatinine; ALB, albumin; TP, total protein; TG, triglycerides; CHOL, cholesterol; LDL, low-density lipoprotein; VLDL, very low-density lipoprotein; HDL, high-density lipoprotein; GLU, glucose; AST, aspartate aminotransferase; ALT, alanine aminotransferase; ALP, alkaline phosphatase; TAC, total antioxidant capacity; GPX, glutathione peroxidase; SOD, superoxide dismutase; MDA, malondialdehyde; *Ca*, calcium; P, phosphorus.

## 4 Discussion

The present study evaluated the effects of RenalMax, a blend of phytogenics and essential nutrients, including nettle extract, sorbitol, ammonium chloride, and vitamins B_1_, B_2_, B_6_, and C, on broiler performance, renal function, lipid metabolism, hepatic enzymes, antioxidant status, and mineral metabolism. However, it is important to distinguish statistical significance from biological relevance. Although some variables, such as dressing percentage, showed statistically significant treatment effects, the numerical differences among treatments were small and are unlikely to have practical importance under commercial production conditions. Therefore, the biological interpretation of the present study was based primarily on the magnitude and consistency of the dose-response relationships described by the regression models rather than on statistical significance alone.

Our findings revealed that RenalMax supplementation significantly enhanced growth performance, modified serum renal- and hepatic-related biochemical markers, altered lipid and glucose profiles, and enhanced antioxidant-related variables. Because RenalMax is a multi-component formulation, the present experiment cannot distinguish the contribution of individual ingredients. Nevertheless, based on previous literature, the observed responses may be associated with the combined biological activities of its phytogenic compounds, vitamins, sorbitol, and ammonium chloride. The nettle extract (*Urtica dioica* L.) is rich in polyphenols such as chlorogenic acid (301 μg/g), caffeic acid (171 μg/g), kaempferol (301 μg/g), and quercetin (303 μg/g), which possess strong antioxidant, anti-inflammatory, and immunostimulatory properties. Sorbitol has been reported to act as an osmotic agent that improves nutrient absorption and reduces early inflammatory responses, contributing to better growth performance and reduced serum glucose and lipid levels (Takahashi et al., 2002). These compounds may contribute to reducing oxidative stress and enhance immune function, contributing to favorable changes in serum biochemical markers associated with renal and hepatic function in broilers (Hashemi & Davoodi, 2010; Ahmadipour & Khajali, 2019).

The results demonstrate significant enhancements in body weight gain and feed intake, indicative of improved growth efficiency and nutrient utilization, aligning with previous findings on phytogenic-nutrient blends (Wang et al., 2021; Al-Garadi et al., 2025). While dressing percentage and liver weight were significantly affected by RenalMax supplementation, the numerical differences in dressing yield were relatively small, suggesting a limited biological impact. Notably, abdominal fat deposition was markedly reduced at the optimal inclusion level, indicating improved lipid metabolism and energy utilization. In contrast, thigh and breast meat yields were not significantly influenced, indicating that RenalMax did not alter muscle deposition patterns. Similarly, heart weight remained unchanged across treatments, suggesting no adverse or stimulatory effects on cardiac development. The reduction in fat pad alongside stable muscle yield suggests a favorable shift in body composition without compromising lean tissue accretion. Notably, RenalMax supplementation reduced serum uric acid, creatinine, and blood urea nitrogen concentrations compared with the control treatment. These changes are consistent with alterations in nitrogen metabolism and serum biochemical markers commonly associated with renal function. Likewise, reductions in AST, ALT, and ALP activities indicate favorable changes in serum biochemical markers associated with hepatic function. However, because the present study did not include histopathological examination, molecular analyses, or functional organ assessments, these findings should not be interpreted as direct evidence of nephroprotection or hepatoprotection (Salih, 2015; Siouda & Abdennour, 2015; Dhouibi et al., 2021; Yu et al., 2022). Liver enzyme activity reductions (AST, ALT, ALP) reflect hepatoprotective effects and reduced oxidative damage, coherent with phytogenics’ antioxidative capacities documented in poultry. Enhanced activities of antioxidant enzymes such as glutathione peroxidase and superoxide dismutase, alongside reduced malondialdehyde, signify strengthened antioxidant defense and diminished oxidative stress, aligning with established roles of supplementation in augmenting cellular redox balance (Surai et al., 2019; Bacou et al., 2021; Abubakar et al., 2023). Finally, marked increases in serum calcium and phosphorus indicate improved mineral absorption and regulation critical for skeletal health and metabolic functions, corroborating findings on mineral metabolism interplay in poultry (Yang et al., 2025). Phytogenics such as nettle extract are rich in bioactive compounds like flavonoids and phenolics, which have been shown to enhance digestive enzyme activity and nutrient absorption (Hashemi and, Davoodi2010). The lack of change in thigh meat yield suggests that RenalMax did not significantly influence protein accretion in muscle tissues. The stable FCR combined with increased intake and gain implies that RenalMax optimizes nutrient utilization without compromising feed efficiency. These results corroborate findings by Rahimi et al. (2011), who reported enhanced broiler growth with phytogenic blends, and reinforce the notion that phytogenic compounds can act synergistically with vitamins to promote growth.

The reductions observed in serum uric acid, creatinine, and blood urea nitrogen suggest altered nitrogen metabolism and changes in serum biochemical variables commonly associated with renal function. These changes may partly reflect biological activities previously reported for nettle extract and other phytogenic compounds (Chrubasik et al., 2007), although the contribution of individual ingredients cannot be distinguished in the present experiment. Ammonium chloride’s role in acidifying urine may have further enhanced renal clearance of nitrogenous wastes, improved nitrogen utilization efficiency and reducing metabolic burden (Horst & Jorgensen, 1974). Lower circulating nitrogenous wastes also likely contribute to improved metabolic status and reduced renal stress, consistent with studies by Abubakar et al. (2023), who demonstrated that phytogenic compounds can ameliorate renal dysfunction markers in poultry.

RenalMax supplementation markedly improved lipid profiles, with substantial decreases in triglycerides, total cholesterol, LDL, and VLDL, alongside a dramatic increase in HDL levels. These findings underscore the lipid-lowering potential of the phytogenic components, particularly flavonoids and vitamin C, which exert hypolipidemic and antioxidant effects by modulating lipid metabolism and preventing oxidative modification of lipoproteins (Kotze, 1975; Domjanic Drozdek et al., 2022). The significant reduction in LDL-cholesterol coupled with HDL elevation is particularly important for cardiovascular health and mirrors outcomes reported by Tehseen et al. (2016) Jang et al. (2008) in broilers supplemented with herbal extracts. Lower glucose concentrations highlight improved carbohydrate metabolism, possibly mediated by vitamin B complex and C, which enhance enzymatic activities critical for glucose utilization (Öztürk Özkan, 2018). Chlorogenic acid, in particular, has been shown to improve growth performance, alleviate oxidative stress, and support hepatic function in broilers by elevating antioxidant enzyme activities and reducing lipid peroxidation (Liang & Kitts, 2015). Both quercetin and kaempferol have been shown to possess strong antioxidant properties. For instance, quercetin oxidation leads to the formation of metabolites that exhibit enhanced antioxidant potency compared to their precursors, indicating that these compounds can effectively protect cells from oxidative stress (Speisky et al., 2023). Additionally, flavonol glucuronide conjugates of quercetin and kaempferol have been identified as contributing to antioxidant effects, which may play a role in reducing lipid peroxidation in cellular membranes (Fernández‐Arroyo et al., 2012).

The pronounced reductions in AST, ALT, and ALP enzyme activities indicate favorable changes in serum enzyme activities commonly associated with hepatic function, likely resulting from its antioxidant-rich phytogenic and vitamin components. These enzymes are sensitive markers of liver injury and oxidative damage; their normalization suggests reduced hepatic stress and improved liver integrity (Samih & Ahami, 2019). The significant decrease in ALP, an enzyme linked to biliary function and liver metabolism, further supports improved hepatic health, consistent with previous studies demonstrating the ability of nettle extract and vitamin supplementation to alleviate liver toxicity and enhance hepatic antioxidant defense (Ramírez-Marroquín & Jiménez-Arellanes, 2019).

RenalMax significantly enhanced antioxidant capacity, demonstrated by increased TAC and elevated activities of GPX and SOD, along with a marked reduction in MDA levels, a key biomarker for lipid peroxidation and oxidative damage. These antioxidant improvements may reflect the combined biological activity of the phytogenic compounds and vitamins present in RenalMax (Sies, 2015; Mnisi et al., 2022). Such data reinforce existing literature on phytogenic antioxidants improving poultry oxidative status and reducing oxidative stress during intensive growth periods (Mohamed & Hassan, 2023). By reducing oxidative damage, RenalMax may contribute to better cellular integrity, immune function, and physiological status.

The significant increase in serum calcium and phosphorus concentrations implies enhanced mineral absorption and metabolic utilization. Vitamins B and C are known to facilitate calcium and phosphorus metabolism through improved intestinal uptake and bone mineralization processes (NRC, 1994). Additionally, ammonium chloride’s metabolic acidifying effect can increase calcium mobilization from bones, optimizing mineral balance (Pearlin et al., 2020). Improved mineral status supports skeletal development and metabolic functions essential for growth, paralleling findings by Flees et al. (2021a) who reported augmented calcium and phosphorus levels following phytogenic supplementation in broilers. Ammonium chloride also plays a role in mineral metabolism by promoting the mobilization of calcium and phosphorus. Research indicates that NH_4_Cl can lead to increased serum ionized calcium concentrations and higher urinary calcium excretion, which are essential for bone health (Ching et al., 1989). This mobilization is vital for the development and maintenance of strong bones in broilers, which is critical for their growth and overall metabolic function. The acidifying effect of ammonium chloride leads to a decrease in urinary pH, which promotes the excretion of hydrogen ions and enhances the clearance of nitrogenous wastes (Edelmann et al., 1967). This mechanism is crucial for maintaining acid-base balance in the body, especially in broilers that may experience metabolic stress.

Dose-response models generally predicted maximal responses within approximately 0.5–0.7 mL/L RenalMax. However, because these estimates were derived from regression analyses based on only four supplementation levels, they should be considered model-based predictions within the experimental range rather than definitive biological optima. This aligns with previous phytogenic research highlighting dose-dependent efficacy with potential plateauing at higher levels (Windisch et al., 2008). The PCA distinguished treatment groups based on metabolic, renal, and growth markers. PC1 was primarily characterized by high positive loadings for lipid-related variables, hepatic enzymes, and MDA together with negative loadings for antioxidant variables and growth traits, whereas PC2 was mainly associated with nitrogen metabolism variables. These multivariate analyses support the observed patterns among measured variables but should not be interpreted as direct evidence of specific physiological processes.

From a practical production perspective, the estimated optimal supplementation range of 0.5–0.6 mL/L should be interpreted as a model-derived response point within the tested range rather than a fixed commercial recommendation. The application value of RenalMax should be further evaluated considering additive cost, farm management conditions, and production objectives. Because RenalMax was administered through drinking water, this approach may provide operational flexibility in commercial broiler production, particularly during growing and finishing periods characterized by rapid growth and increased metabolic demands. However, further studies are required to evaluate its long-term safety, economic return, and applicability during different production phases, including the starter and brooding periods.

## 5 Conclusion

RenalMax supplementation modified growth performance, serum biochemical variables, antioxidant indices, and mineral-related variables in broiler chickens. Collectively, these responses indicate that the formulation may favorably influence several physiological processes associated with growth, lipid metabolism, oxidative status, and nitrogen metabolism. Dose-response modeling generally identified maximal predicted responses between approximately 0.5 and 0.6 mL/L; however, these values should be interpreted cautiously because they were derived from regression models based on only four supplementation levels. Additional studies incorporating more dose levels together with histopathological and molecular assessments are warranted to validate these findings and confirm the underlying biological mechanisms.

## Ethical approval

All experimental procedures involving birds were conducted in accordance with the guidelines of the Iranian Council of Animal Care (ICAC) for the ethical use of animals in research. The study protocol, including housing, management, handling, sampling, and euthanasia procedures, was reviewed and approved by the Research Animal Ethics Committee of the University of Zabol, Iran (Approval Code: AEUOZ-2012 | UP-2020-BR-21).

## Animal welfare

Throughout the 42-day experiment, birds were monitored daily for health, behavior, and welfare. Stocking density, temperature, ventilation, lighting, and access to feed and water were maintained according to standard broiler management guidelines. Any bird showing signs of severe distress or illness was humanely euthanized following the committee-approved procedures.

## Compliance

This study fully complies with national and institutional guidelines for the care and use of animals in research. No unnecessary stress or discomfort was imposed on the animals, and all efforts were made to minimize pain or distress (Fig. 1, Fig. 2, Fig. 3, Fig. 4, Fig. 5, Fig. 6).

**Fig. 1:**
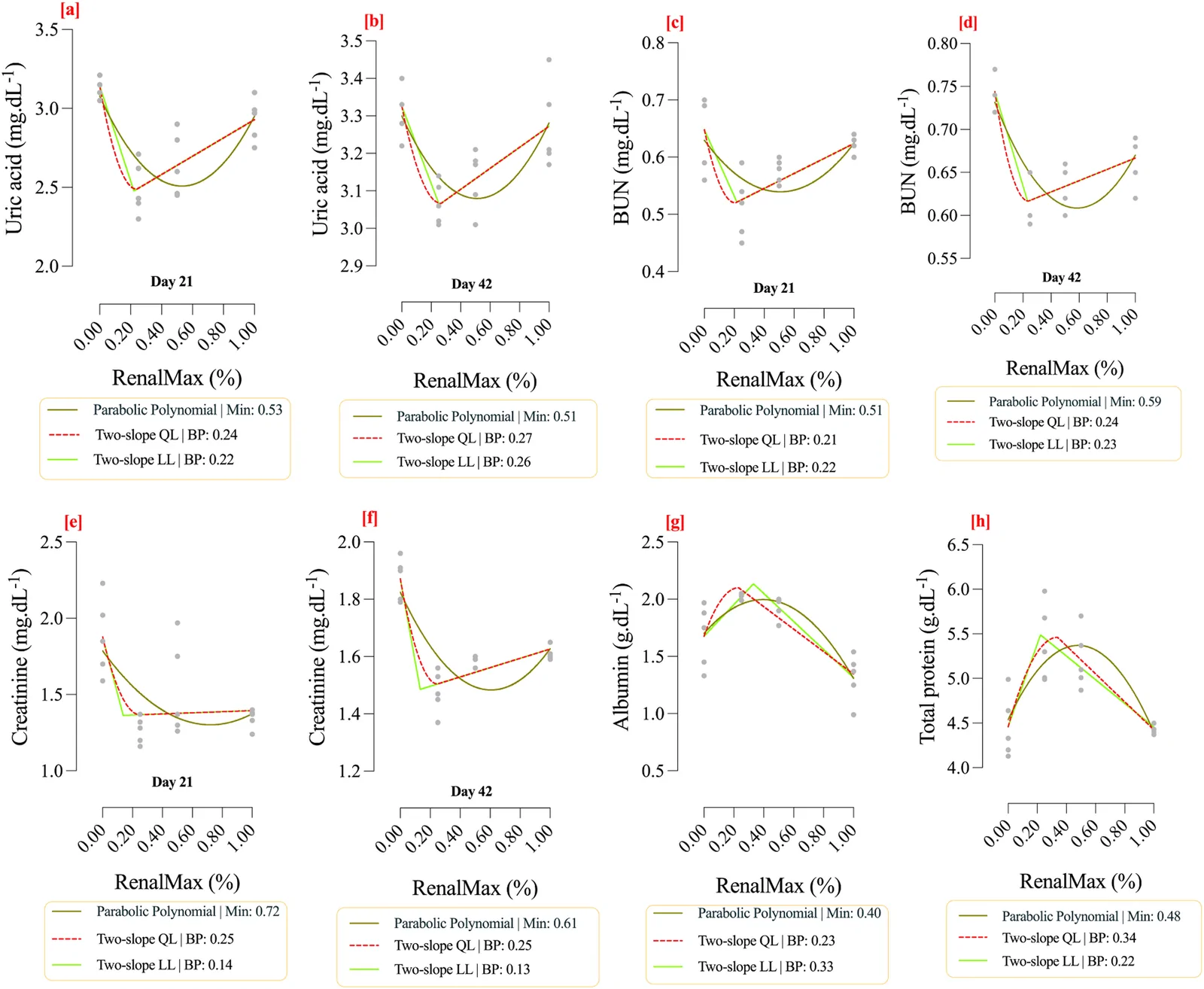
Dose–response relationships between RenalMax (%) and notrogen compounds in serum. [a]: uric acid at d 21, [b]: uric acid at d 42, [c and d]: blood urea nitrogen at d 21 and 42 (BUN), [e]: cratinine at d 21, [f]: cratinine at d 42, [g]: albumin, and [h]: total protein. Data points represent individual values, while lines show model fits including parabolic polynomial, two-slope linear, and two-slope quadratic-linear.

**Fig. 2:**
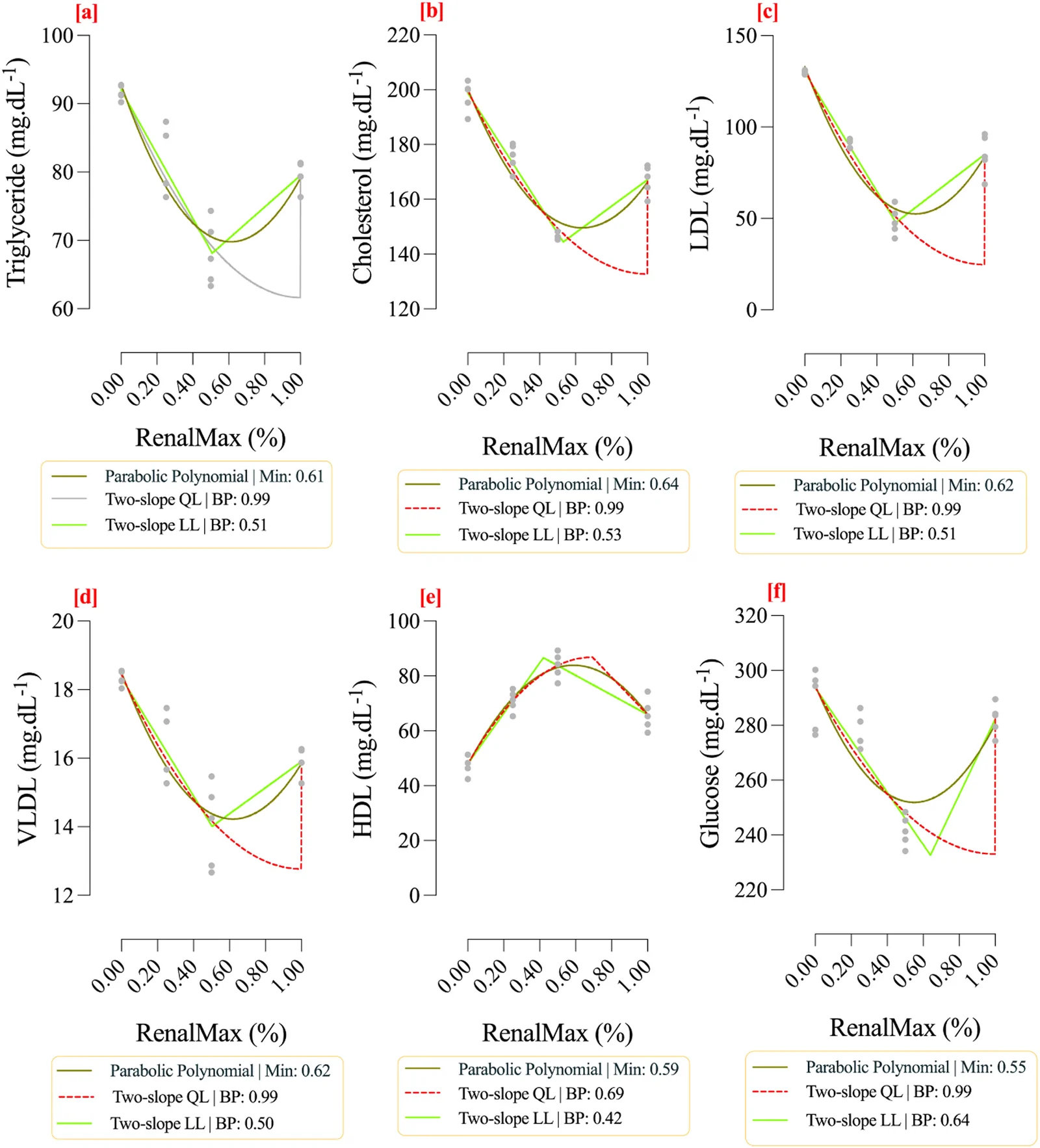
Dose–response relationships between RenalMax (%) and lipid constituates and glucose in serum. [a]: triglycerides, [b]: cholesterol, [c]: low-density lipoprotein (LDL), [d]: very low-density lipoprotein (VLDL), [e]: high-density lipoprotein (HDL), and [f]: glucose. Data points represent individual values, while lines show model fits including parabolic polynomial, two-slope linear, and two-slope quadratic-linear.

**Fig. 3:**
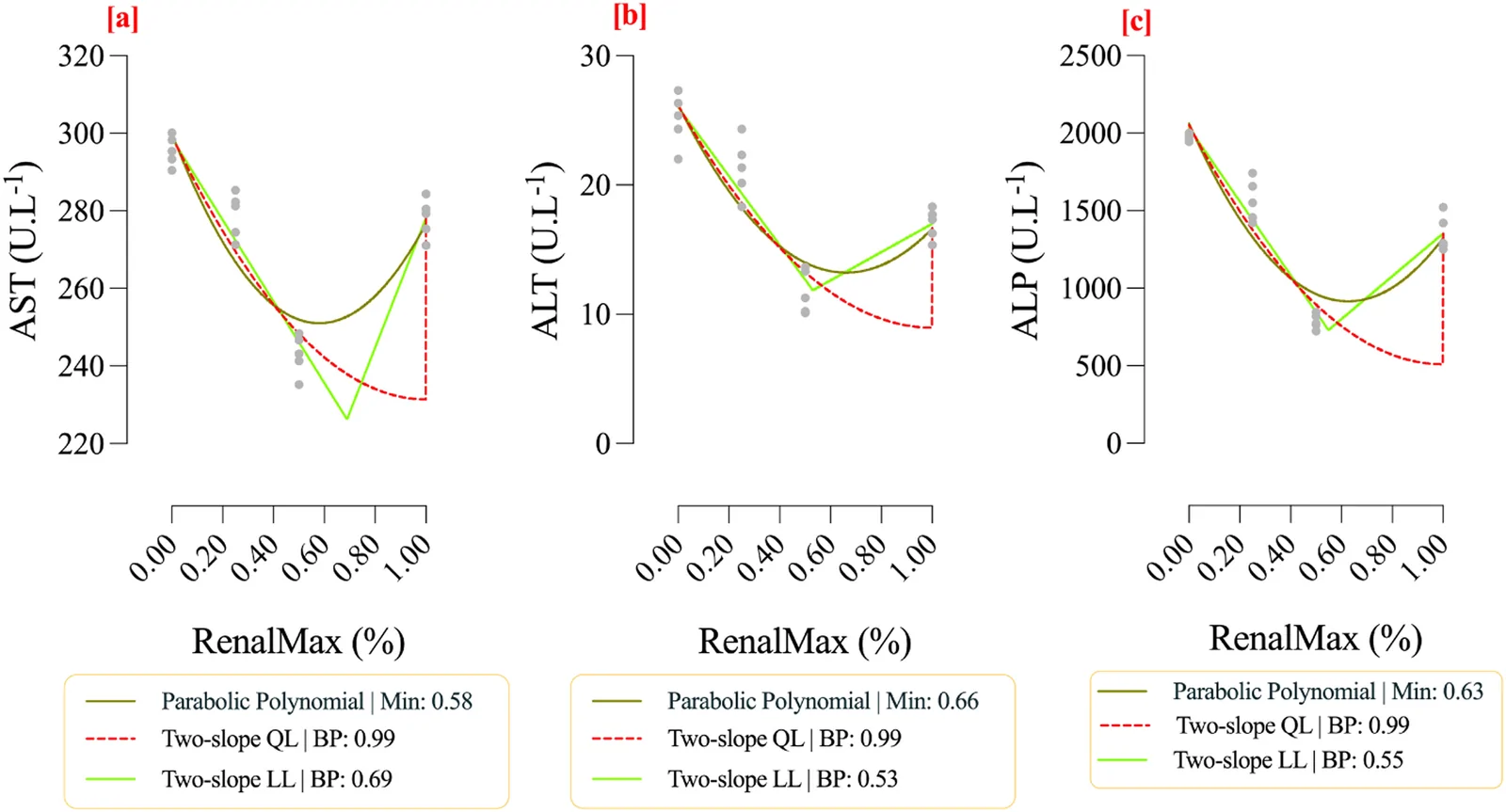
Dose–response relationships between RenalMax (%) and hepatic enzyme activities in sera. [a]: aspartate aminotransferase (AST), [b]: alanine aminotransferase (ALT), and [c]: alkaline phosphatase (ALP). Data points represent individual values, while lines show model fits including parabolic polynomial, two-slope linear, and two-slope quadratic-linear.

**Fig. 4:**
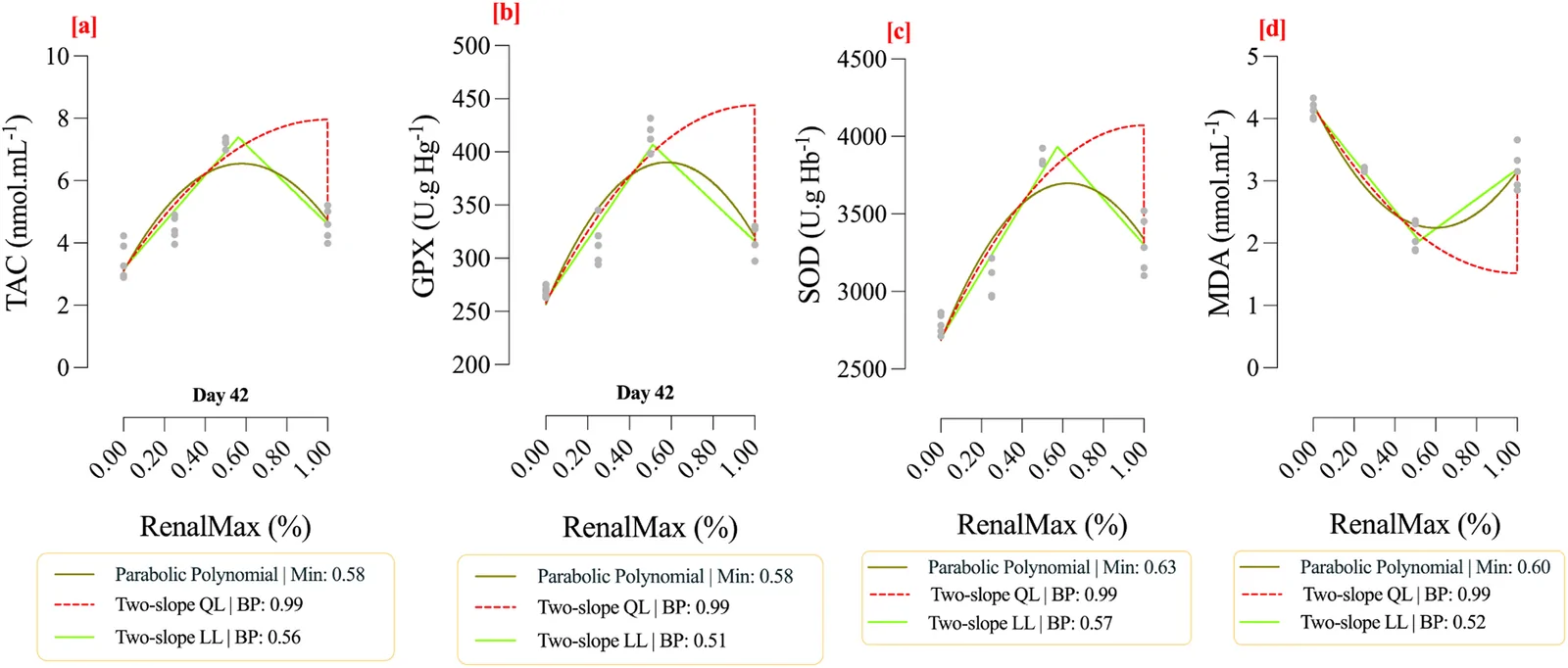
Dose–response relationships between RenalMax (%) and antioxidant system. [a]: total antioxidant capacity (TAC), [b]: gluthatione peroxidase (GPX), [c]: superoxide dismutase (SOD), and [d]: malondialdehyde (MDA). Data points represent individual values, while lines show model fits including parabolic polynomial, two-slope linear, and two-slope quadratic-linear.

**Fig. 5:**
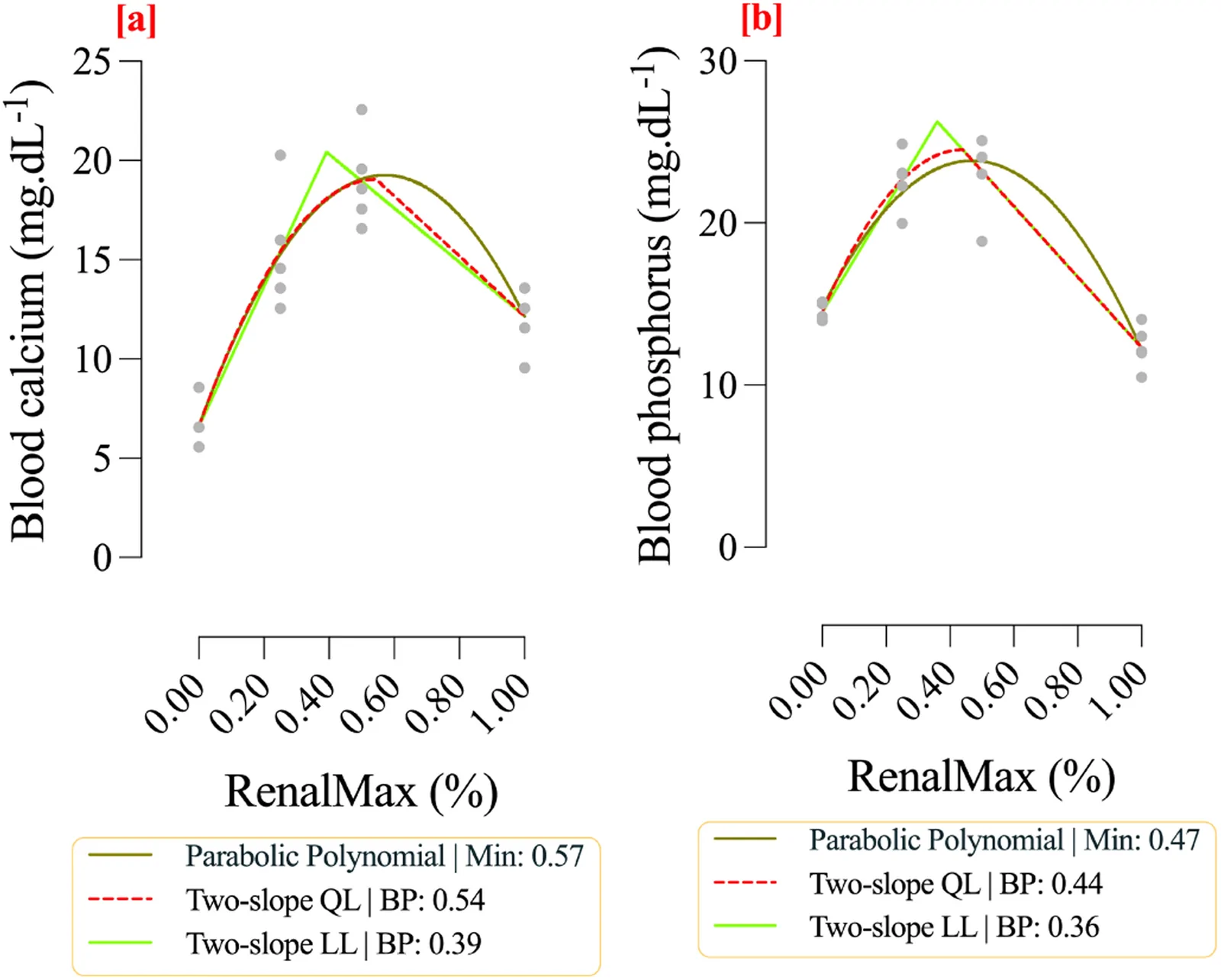
Dose–response relationships between RenalMax (%) and calcium and phosphorus in serum. [a]: blood calcium and [b]: blood phosphrus. Data points represent individual values, while lines show model fits including parabolic polynomial, two-slope linear, and two-slope quadratic-linear.

**Fig. 6:**
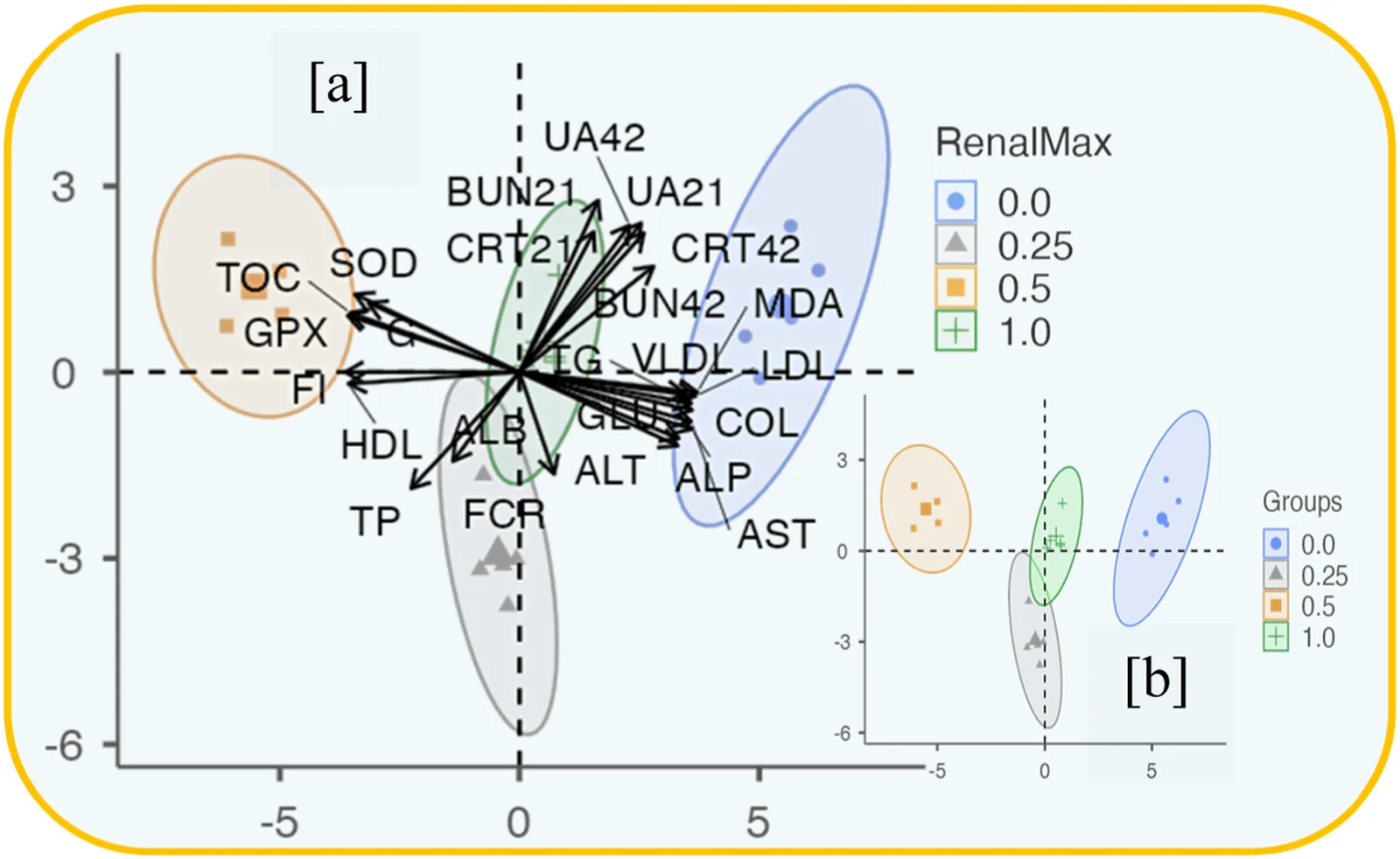
Principal component analysis (PCA) biplot illustrating the effects of RenalMax supplementation (0.0, 0.25, 0.5, and 1.0%) on serum biochemical, antioxidant, and metabolic variables. (a) Variable loadings show associations among enzymes, metabolites, and antioxidant markers, with arrows indicating direction and strength of contribution to group separation. (b) Score plot displays group clustering by RenalMax level, highlighting clear separation between treatments. UA: uric acid (d 21 and 42), CRT: creatinine (d 21 and 42), BUN: blood urea nitrogen (d 21 and 42), ALB: albumin, TP: total protein, TG: triglyceride, VLDL: very low-density lipoprotein, HDL: high-density lipoprotein, LDL: low-density lipoprotein, COL: cholesterol, GLU: glucose, AST: aspartate transaminase, ALT: alanine transaminase, ALP: alkaline phosphatase, TOC: total antioxidant capacity, GPX: gluthatione peroxidase, SOD: superoxide dismutase, MDA: amalodialdehyde, FI: feed intake, G: body weight, FCR: feed conversion ratio.

## Funding statement

This study was supported by internal funding provided by Makian Rooyesh Darou Co.

## CRediT authorship contribution statement

**Morteza Asghari-Moghadam:** Writing – review & editing, Supervision, Project administration, Methodology, Investigation, Conceptualization. **Hamid-Reza Behboodi:** Writing – review & editing, Visualization, Validation, Investigation, Data curation. **Mehran Mehri:** Writing – review & editing, Writing – original draft, Visualization, Validation, Software, Formal analysis.

## Declaration of competing interest

The authors declare that they have no known competing financial interests or personal relationships that could have appeared to influence the work reported in this paper.

## Data Availability

The data presented in this study are available from the corresponding author upon reasonable request.
